# Correction: Proximate and distant determinants of maternal and neonatal mortality in the postnatal period: A scoping review of data from low- and middle-income countries

**DOI:** 10.1371/journal.pone.0304260

**Published:** 2024-05-20

**Authors:** The PLOS One Staff

There are errors in the author affiliations. The correct affiliations are as follows:

Preston Izulla^1^, Angela Muriuki^2^, Michael Kiragu^1^, Melanie Yahner^4^, Virginia Fonner^1,5^, Syeda Nabin Ara Nitu^3^, Bernard Osir^1^, Farahat Bello^4^, Joseph de Graft-Johnson^4^

**1** Adroitz Consultants Limited, Nairobi, Kenya, **2** Save the Children, Kenya Regional Office, Nairobi, Kenya, **3** Save the Children, Dhaka, Bangladesh, **4** Save the Children, Washington DC, United States,

**5** The Medical University of South Carolina, South Carolina, United States

The publisher apologizes for the error.
