# Supplementary figures and images for: Proximate and distant determinants of maternal and neonatal mortality in the postnatal period: A scoping review of data from low- and middle-income countries
Source: PLoS One. 2023 Nov 20;18(11):e0293479. doi: 10.1371/journal.pone.0293479 (PMC10659187; doi:10.1371/journal.pone.0293479)

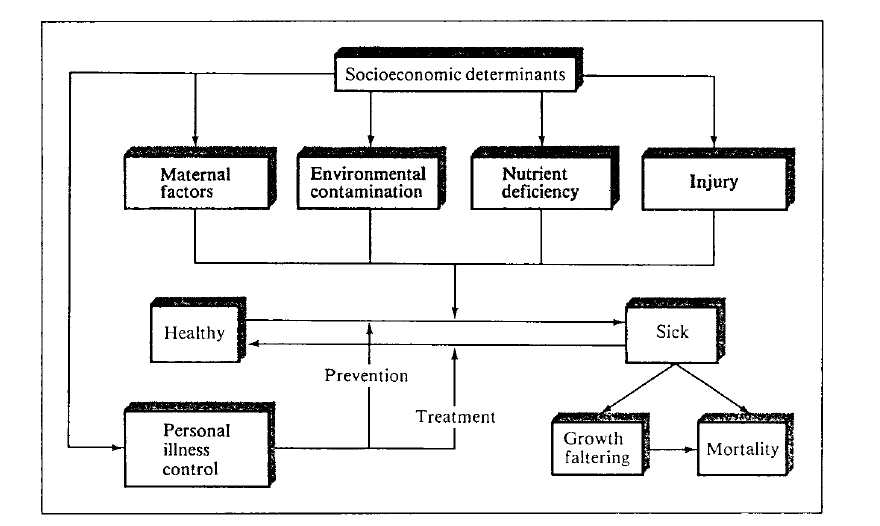

Supplement: S1 Fig — (TIF) [file pone.0293479.s002.tif]
