# Supplementary material for: Proximate and distant determinants of maternal and neonatal mortality in the postnatal period: A scoping review of data from low- and middle-income countries
Source: PLoS One. 2023 Nov 20;18(11):e0293479. doi: 10.1371/journal.pone.0293479 (PMC10659187; doi:10.1371/journal.pone.0293479)
Supplement: S1 File — (DOCX) [file pone.0293479.s003.docx]

**Supplemental File 1- Scoping Review Search Terms**

**PubMed**

(((("maternal mortality"[MeSH] OR ("maternal"[All Fields] AND "mortality"[All Fields]) OR "maternal mortality"[All Fields] OR "Infant Mortality"[MeSH] OR "perinatal death"[MeSH] or "infant death"[MeSH] OR "maternal death"[tiab] OR "newborn death"[tiab] OR "neonatal death"[tiab])) AND ((("Risk factors"[MeSH] OR "Epidemiologic studies"[MeSH] OR "Odds ratio"[MeSH] OR "Multivariate analysis"[MeSH] OR "Logistic Models"[MeSH] OR "Prevalence"[MeSH] OR "Incidence"[MeSH] OR "odds ratio"[tiab] OR "risk ratio"[tiab] OR "relative risk"[tiab] OR "risk factor"[tiab] OR risk[tiab] OR predict*[tiab] OR correlat*[tiab] OR etiol*[tiab] OR aetiol*[tiab] OR prevalence[tiab] OR incidence[tiab] OR rate*[tiab])))) AND ((afghanistan[MeSH] OR albania[MeSH] OR algeria[MeSH] OR american samoa[MeSH] OR angola[MeSH] OR antigua and barbuda[MeSH] OR argentina[MeSH] OR armenia[MeSH] OR aruba[MeSH] OR azerbaijan[MeSH] OR bahrain[MeSH] OR bangladesh[MeSH] OR barbados[MeSH] OR republic of belarus[MeSH] OR belize[MeSH] OR benin[MeSH] OR bhutan[MeSH] OR bolivia[MeSH] OR bosnia and herzegovina[MeSH] OR botswana[MeSH] OR brazil[MeSH] OR bulgaria[MeSH] OR burkina faso[MeSH] OR burundi[MeSH] OR cabo verde[MeSH] OR cambodia[MeSH] OR cameroon[MeSH] OR central african republic[MeSH] OR chad[MeSH] OR chile[MeSH] OR china[MeSH] OR colombia[MeSH] OR comoros[MeSH] OR democratic republic of the congo[MeSH] OR congo[MeSH] OR costa rica[MeSH] OR cote d’ivoire[MeSH] OR croatia[MeSH] OR cuba[MeSH] OR cyprus[MeSH] OR czech republic[MeSH] OR djibouti[MeSH] OR dominica[MeSH] OR dominican republic[MeSH] OR ecuador[MeSH] OR egypt[MeSH] OR el salvador[MeSH] OR equatorial guinea[MeSH] OR eritrea[MeSH] OR estonia[MeSH] OR swaziland[MeSH] OR ethiopia[MeSH] OR fiji[MeSH] OR gabon[MeSH] OR gambia[MeSH] OR georgia (republic)[MeSH] OR ghana[MeSH] OR gibraltar[MeSH] OR greece[MeSH] OR grenada[MeSH] OR guam[MeSH] OR guatemala[MeSH] OR guinea[MeSH] OR guinea bissau[MeSH] OR guyana[MeSH] OR haiti[MeSH] OR honduras[MeSH] OR hungary[MeSH] OR india[MeSH] OR indonesia[MeSH] OR iran[MeSH] OR iraq[MeSH] OR jamaica[MeSH] OR jordan[MeSH] OR kazakhstan[MeSH] OR kenya[MeSH] OR democratic people’s republic of korea[MeSH] OR republic of korea[MeSH] OR kosovo[MeSH] OR kyrgyzstan[MeSH] OR laos[MeSH] OR latvia[MeSH] OR lebanon[MeSH] OR lesotho[MeSH] OR liberia[MeSH] OR libya[MeSH] OR lithuania[MeSH] OR macau[MeSH] OR republic of north macedonia[MeSH] OR madagascar[MeSH] OR malawi[MeSH] OR malaysia[MeSH] OR indian ocean islands[MeSH] OR mali[MeSH] OR malta[MeSH] OR micronesia[MeSH] OR palau[MeSH] OR mauritania[MeSH] OR mauritius[MeSH] OR mexico[MeSH] OR moldova[MeSH] OR mongolia[MeSH] OR montenegro[MeSH] OR morocco[MeSH] OR mozambique[MeSH] OR myanmar[MeSH] OR namibia[MeSH] OR nepal[MeSH] OR netherlands antilles[MeSH] OR nicaragua[MeSH] OR niger[MeSH] OR nigeria[MeSH] OR oman[MeSH] OR pakistan[MeSH] OR panama[MeSH] OR papua new guinea[MeSH] OR paraguay[MeSH] OR peru[MeSH] OR philippines[MeSH] OR poland[MeSH] OR portugal[MeSH] OR puerto rico[MeSH] OR romania[MeSH] OR russia[MeSH] OR rwanda[MeSH] OR samoa[MeSH] OR sao tome and principe[MeSH] OR saudi arabia[MeSH] OR senegal[MeSH] OR serbia[MeSH] OR seychelles[MeSH] OR sierra leone[MeSH] OR slovakia[MeSH] OR slovenia[MeSH] OR melanesia[MeSH] OR somalia[MeSH] OR south africa[MeSH] OR south sudan[MeSH] OR sri lanka[MeSH] OR saint kitts and nevis[MeSH] OR saint lucia[MeSH] OR saint vincent and the grenadines[MeSH] OR sudan[MeSH] OR suriname[MeSH] OR syria[MeSH] OR tajikistan[MeSH] OR tanzania[MeSH] OR thailand[MeSH] OR timor leste[MeSH] OR togo[MeSH] OR tonga[MeSH] OR trinidad and tobago[MeSH] OR tunisia[MeSH] OR turkey[MeSH] OR turkmenistan[MeSH] OR uganda[MeSH] OR ukraine[MeSH] OR uruguay[MeSH] OR uzbekistan[MeSH] OR vanuatu[MeSH] OR venezuela[MeSH] OR vietnam[MeSH] OR middle east[MeSH] OR yemen[MeSH] OR yugoslavia[MeSH] OR zambia[MeSH] OR zimbabwe[MeSH] OR africa south of the sahara[MeSH] OR africa, central[MeSH] OR africa, northern[MeSH] OR africa, southern[MeSH] OR africa, eastern[MeSH] OR africa, western[MeSH] OR west indies[MeSH] OR indian ocean islands[MeSH] OR caribbean region[MeSH] OR central america[MeSH] OR latin america[MeSH] OR south america[MeSH] OR asia, central[MeSH] OR asia, northern[MeSH] OR asia, southeastern[MeSH] OR asia, western[MeSH] OR europe, eastern[MeSH] OR "developing countries"[MeSH]) OR "developing country"[tiab] OR "developing countries"[tiab] OR "developing nation"[tiab] OR "developing nations"[tiab] OR "middle income country"[tiab] OR "middle income countries"[tiab] OR "low income country"[tiab] OR "low income countries"[tiab] OR "low and middle income countries"[tiab]))) AND (Puerperium [MeSH] or postnatal[tiab] or "post natal"[tiab] or "post-partum"[tiab] or postpartum[tiab] or parturition[tiab] or perinatal[tiab] or peripartum[tiab] or intrapartum[tiab]) AND (2011:2022[pdat])

**Scopus**

(TITLE-ABS-KEY( "maternal mortality" OR "maternal death" OR "newborn death" OR "neonatal death" OR "neonatal mortality" OR "newborn mortality" OR "Infant Mortality" OR "perinatal death" OR "perinatal mortality" OR "infant death" )) AND (("Risk factors" OR "Epidemiologic studies" OR "Odds ratio" OR "Multivariate analysis" OR "Logistic Models" OR "Prevalence" OR "Incidence" OR "odds ratio" OR "risk ratio" OR "relative risk" OR "risk factor" OR risk OR predict* OR correlat* OR etiol* OR aetiol* OR prevalence OR incidence OR rate*)) AND (afghanistan OR albania OR algeria OR "american samoa" OR angola OR "antigua and Barbuda" OR argentina OR armenia OR aruba OR azerbaijan OR bahrain OR bangladesh OR barbados OR republic of belarus OR belize OR benin OR bhutan OR bolivia OR "bosnia and Herzegovina" OR botswana OR brazil OR bulgaria OR "burkina faso" OR burundi OR "cabo verde" OR cambodia OR cameroon OR "central african republic" OR chad OR chile OR china OR colombia OR comoros OR congo OR "costa rica" OR "cote d'ivoire" OR croatia OR cuba OR cyprus OR "czech republic" OR djibouti OR dominica OR "dominican republic" OR ecuador OR egypt OR "el Salvador" OR "equatorial guinea" OR eritrea OR estonia OR swaziland OR eswatini OR ethiopia OR fiji OR gabon OR gambia OR georgia OR ghana OR gibraltar OR greece OR grenada OR guam OR guatemala OR guinea OR "guinea Bissau" OR guyana OR haiti OR honduras OR hungary OR india OR indonesia OR iran OR iraq OR jamaica OR jordan OR kazakhstan OR kenya OR korea OR kosovo OR kyrgyzstan OR laos OR latvia OR lebanon OR lesotho OR liberia OR libya OR lithuania OR macau OR macedonia OR madagascar OR malawi OR malaysia OR "indian ocean islands" OR mali OR malta OR micronesia OR palau OR mauritania OR mauritius OR mexico OR moldova OR mongolia OR montenegro OR morocco OR mozambique OR myanmar OR namibia OR nepal OR "netherlands antilles" OR nicaragua OR niger OR nigeria OR oman OR pakistan OR panama OR "papua new guinea" OR paraguay OR peru OR philippines OR poland OR portugal OR "puerto rico" OR romania OR russia OR rwanda OR samoa OR "sao tome and principe" OR "saudi arabia" OR senegal OR serbia OR seychelles OR "sierra leone" OR slovakia OR slovenia OR melanesia OR somalia OR "south Africa" OR "south sudan" OR "sri lanka" OR "saint kitts and nevis" OR "saint lucia" OR "saint vincent and the grenadines" OR sudan OR suriname OR syria OR tajikistan OR tanzania OR thailand OR "timor leste" OR togo OR tonga OR "trinidad and tobago" OR tunisia OR turkey OR turkmenistan OR uganda OR ukraine OR uruguay OR uzbekistan OR vanuatu OR venezuela OR vietnam OR "middle east" OR yemen OR yugoslavia OR zambia OR zimbabwe OR africa OR "west indies" OR "indian ocean islands" OR "caribbean region" OR "central America" OR "latin America" OR "south America" OR asia OR "eastern euopre" OR "developing countries" OR "developing country" OR "developing countries" OR "developing nation" OR "developing nations" OR "middle income country" OR "middle income countries" OR "low income country" OR "low income countries" OR "low and middle income countries") AND (Puerperium OR postnatal OR postpartum OR parturition OR perinatal OR peripartum OR intrapartum) AND ( LIMIT-TO ( PUBYEAR,2022) OR LIMIT-TO ( PUBYEAR,2021) OR LIMIT-TO ( PUBYEAR,2020) OR LIMIT-TO ( PUBYEAR,2019) OR LIMIT-TO ( PUBYEAR,2018) OR LIMIT-TO ( PUBYEAR,2017) OR LIMIT-TO ( PUBYEAR,2016) OR LIMIT-TO ( PUBYEAR,2015) OR LIMIT-TO ( PUBYEAR,2014) OR LIMIT-TO ( PUBYEAR,2013) OR LIMIT-TO ( PUBYEAR,2012) OR LIMIT-TO ( PUBYEAR,2011) )

**CINAHL and PsycINFO (same terms used for each database)**

( ( "maternal mortality" OR "maternal death" OR "newborn death" OR "neonatal death" OR "neonatal mortality" OR "newborn mortality" OR "Infant Mortality" OR "perinatal death" OR "perinatal mortality" OR "infant death" ) ) AND ( (“Risk factors" OR "Epidemiologic studies" OR "Odds ratio" OR "Multivariate analysis" OR "Logistic Models" OR "Prevalence" OR "Incidence" OR "odds ratio" OR "risk ratio" OR "relative risk" OR "risk factor" OR risk OR predict* OR correlat* OR etiol* OR aetiol* OR prevalence OR incidence OR rate*) ) AND ( (Puerperium OR postnatal OR postpartum OR parturition OR perinatal OR peripartum OR intrapartum) ) AND ( (afghanistan OR albania OR algeria OR “american samoa” OR angola OR “antigua and Barbuda” OR argentina OR armenia OR aruba OR azerbaijan OR bahrain OR bangladesh OR barbados OR republic of belarus OR belize OR benin OR bhutan OR bolivia OR “bosnia and Herzegovina” OR botswana OR brazil OR bulgaria OR “burkina faso” OR burundi OR “cabo verde” OR cambodia OR cameroon OR “central african republic” OR chad OR chile OR china OR colombia OR comoros OR congo OR “costa rica” OR “cote d’ivoire” OR croatia OR cuba OR cyprus OR “czech republic” OR djibouti OR dominica OR “dominican republic” OR ecuador OR egypt OR “el Salvador” OR “equatorial guinea” OR eritrea OR estonia OR swaziland OR eswatini OR ethiopia OR fiji OR gabon OR gambia OR georgia OR ghana OR gibraltar OR greece OR grenada OR guam OR guatemala OR guinea OR “guinea Bissau” OR guyana OR haiti OR honduras OR hungary OR india OR indonesia OR iran OR iraq OR jamaica OR jordan OR kazakhstan OR kenya OR korea OR kosovo OR kyrgyzstan OR laos OR latvia OR lebanon OR lesotho OR liberia OR libya OR lithuania OR macau OR macedonia OR madagascar OR malawi OR malaysia OR “indian ocean islands” OR mali OR malta OR micronesia OR palau OR mauritania OR mauritius OR mexico OR moldova OR mongolia OR montenegro OR morocco OR mozambique OR myanmar OR namibia OR nepal OR “netherlands antilles” OR nicaragua OR niger OR nigeria OR oman OR pakistan OR panama OR “papua new guinea” OR paraguay OR peru OR philippines OR poland OR portugal OR “puerto rico” OR romania OR russia OR rwanda OR samoa OR “sao tome and principe” OR “saudi arabia” OR senegal OR serbia OR seychelles OR “sierra leone” OR slovakia OR slovenia OR melanesia OR somalia OR “south Africa” OR “south sudan” OR “sri lanka” OR “saint kitts and nevis” OR “saint lucia” OR “saint vincent and the grenadines” OR sudan OR suriname OR syria OR tajikistan OR tanzania OR thailand OR “timor leste” OR togo OR tonga OR “trinidad and tobago” OR tunisia OR turkey OR turkmenistan OR uganda OR ukraine OR uruguay OR uzbekistan OR vanuatu OR venezuela OR vietnam OR “middle east” OR yemen OR yugoslavia OR zambia OR zimbabwe OR africa OR “west indies” OR “indian ocean islands” OR “caribbean region” OR “central America” OR “latin America” OR “south America” OR asia OR “eastern euopre” OR “developing countries” OR “developing country” OR “developing countries” OR “developing nation” OR “developing nations” OR “middle income country” OR “middle income countries” OR “low income country” OR “low income countries” OR “low and middle income countries”) )

Citations restricted to 2011-2022 dates
