# Supplementary material for: Proximate and distant determinants of maternal and neonatal mortality in the postnatal period: A scoping review of data from low- and middle-income countries
Source: PLoS One. 2023 Nov 20;18(11):e0293479. doi: 10.1371/journal.pone.0293479 (PMC10659187; doi:10.1371/journal.pone.0293479)
Supplement: S2 File — (DOCX) [file pone.0293479.s004.docx]

Risk of mortality among mothers and newborns during the postnatal period. A systematic review of evidence from low- and middle-income countries

To enable PROSPERO to focus on COVID-19 submissions, this registration record has undergone basic automated checks for eligibility and is published exactly as submitted. PROSPERO has never provided peer review, and usual checking by the PROSPERO team does not endorse content. Therefore, automatically published records should be treated as any other PROSPERO registration. Further detail is provided [here](https://www.crd.york.ac.uk/prospero/documents/PROSPEROLetterForAutoPublishJournalRejects.pdf).

Citation

Michael Kiragu, Preston Izulla, Virginia Fonner, Angela Muriuki, Joseph de Graft-Johnson, Melanie Yahner. Risk of mortality among mothers and newborns during the postnatal period. A systematic review of evidence from low- and middle-income countries. PROSPERO 2021 CRD42021234905 Available from: <https://www.crd.york.ac.uk/prospero/display_record.php?ID=CRD42021234905>

Review question

What risk factors are associated with postnatal mortality among mothers and neonates in low- and middle-income countries (LMIC)?

Searches

Publications will be identified through electronic searches of bibliographic databases and grey literature sites, examining citations of retrieved studies, and contacting researchers working in the area. Bibliographic databases to be searched are: PubMed, Scopus, CINAHL and PsycINFO. Specific MeSH and keywords will be modified for use in the databases searched. Trained research assistants will conduct an initial screening of all citations identified in the database search, and will exclude studies not relevant to risk of maternal and neonatal mortality in the postnatal period. Two reviewers (PI and MK) will then independently screen all remaining citations and will categorize studies as eligible for inclusion, not eligible for inclusion, or questionable. Discrepancies in categorization will be resolved through consensus among the reviewers and in cases where consensus will not be reached, a third reviewer (VF), will support decision making for categorization.

Relevant grey literature will be identified from grey literature databases, direct searches of pre-identified organizational and scientific conference websites. We will use advanced search syntax in Google along with a range of combination of search terms including to identify grey literature meeting our inclusion criteria. We will search the following grey literature databases: New York Academy of Medicine-Grey Literature Report, OpenGrey and GreyNet. In addition, key organizational and conference websites will also be searched for reports, conference abstracts, doctoral dissertations and other materials that may have evidence from programme work.

Trained research assistants will search organizational and conference websites for relevant documents. Two senior reviewers (PI and MK) will independently review documents obtained from the grey literature search to ascertain eligibility, with discrepancies resolved through consensus and if necessary, through input from the third reviewer (VF). We will limit our search to documents in English language or those with available English translations published from 1st January 2011 to 31st December 2022.

Search strategy

<https://www.crd.york.ac.uk/PROSPEROFILES/234905_STRATEGY_20210203.pdf>
