# Supplementary material for: Proximate and distant determinants of maternal and neonatal mortality in the postnatal period: A scoping review of data from low- and middle-income countries
Source: PLoS One. 2023 Nov 20;18(11):e0293479. doi: 10.1371/journal.pone.0293479 (PMC10659187; doi:10.1371/journal.pone.0293479)
Supplement: Attachment — Submitted filename: Reviewer coments on scoping review.docx [file pone.0293479.s005.docx]

**Proximate and distant determinants of maternal and neonatal mortality in the postnatal period: A scoping review of data from low- and middle-income countries**

**General comments:**

- References are missing in the results section. For examples, where facts are produced from various studies reported under Proximate and distant factors.

**Comments:**

- At line 90-92, there is a repetition of point. Please delete.
- In the methods section, please indicate whether a review protocol exists. If available, please provide registration details, including registration number.
- Present the full electronic search strategy for at least one database, including any limits used in the methods section.
- Methodological Quality assessment is not clearly mentioned. Authors are requested to elaborate in this part of methods.
- Time line mentioned in the methodology and supplementary material are different. Kindly clarify.
- Multiple gestation may come under Maternal factors but not under neonatal factors. Authors are requested to relook into this particular variable.
- Since authors have included multiple study designs, it is suggested to categorize number of studies included and reported (E.g. no of analytical cross-sectionals studies included).
